# Development of the Australian Cancer Atlas: spatial modelling, visualisation, and reporting of estimates

**DOI:** 10.1186/s12942-019-0185-9

**Published:** 2019-10-01

**Authors:** Earl W. Duncan, Susanna M. Cramb, Joanne F. Aitken, Kerrie L. Mengersen, Peter D. Baade

**Affiliations:** 1 ARC Centre of Excellence for Mathematical and Statistical Frontiers, Queensland University of Technology (QUT), Brisbane, Australia; 2 School of Mathematics, Science and Engineering Faculty, Queensland University of Technology (QUT), Brisbane, Australia; 3 Cancer Council Queensland, PO Box 201, Spring Hill, Brisbane, QLD 4004 Australia; 4 Menzies Health Institute Queensland, Griffith University, Gold Coast, Australia; 5 School of Public Health, The University of Queensland, Brisbane, Australia; 6 School of Research-Public Health, Queensland University of Technology, Brisbane, Australia; 7 Institute for Resilient Regions, University of Southern Queensland, Brisbane, Australia

**Keywords:** Australian Cancer Atlas, Cancer incidence, Relative survival, Spatial smoothing

## Abstract

**Background:**

It is well known that the burden caused by cancer can vary geographically, which may relate to differences in health, economics or lifestyle. However, to date, there was no comprehensive picture of how the cancer burden, measured by cancer incidence and survival, varied by small geographical area across Australia.

**Methods:**

The Atlas consists of 2148 Statistical Areas level 2 across Australia defined by the Australian Statistical Geography Standard which provide the best compromise between small population and small area. Cancer burden was estimated for males, females, and persons separately, with 50 unique sex-specific (males, females, all persons) cancer types analysed. Incidence and relative survival were modelled with Bayesian spatial models using the Leroux prior which was carefully selected to provide adequate spatial smoothing while reflecting genuine geographic variation. Markov Chain Monte Carlo estimation was used because it facilitates quantifying the uncertainty of the posterior estimates numerically and visually.

**Results:**

The results of the statistical model and visualisation development were published through the release of the Australian Cancer Atlas (https://atlas.cancer.org.au) in September, 2018. The Australian Cancer Atlas provides the first freely available, digital, interactive picture of cancer incidence and survival at the small geographical level across Australia with a focus on incorporating uncertainty, while also providing the tools necessary for accurate estimation and appropriate interpretation and decision making.

**Conclusions:**

The success of the Atlas will be measured by how widely it is used by key stakeholders to guide research and inform decision making. It is hoped that the Atlas and the methodology behind it motivates new research opportunities that lead to improvements in our understanding of the geographical patterns of cancer burden, possible causes or risk factors, and the reasons for differences in variation between cancer types, both within Australia and globally. Future versions of the Atlas are planned to include new data sources to include indicators such as cancer screening and treatment, and extensions to the statistical methods to incorporate changes in geographical patterns over time.

## Background

There is a long history of studies showing that where you live matters [1–5]. This can relate to health, economics or lifestyle. It is no different with cancer. The existence of significant geographic variation in cancer incidence and mortality is well known [6, 7], however, most analyses until now have been based on relatively broad or socioeconomically heterogenous geographic areas, precluding detailed area-based comparisons. A number of health-related and cancer-specific interactive, online atlases have been previously released in other countries, including, for example, the Environmental Health Atlas (UK), the National Cancer Institute Cancer Atlas (USA), The Centre for Disease Control Interactive Cancer Atlas (USA), and cancer mortality maps in Spain by province and municipal level [8–11]. However to date, there has been no comprehensive atlas of cancer in Australia.

The Australian Cancer Atlas [12], launched in September 2018, provides the first digital, interactive picture of cancer burden at the small geographical level across Australia, including modelled estimates of both cancer incidence and survival, and incorporating the statistical methodology and visualisation methods that are required for accurate estimation, interpretation and decision making.

Given the increasing number of cancer atlases available internationally, the variety of statistical methods and visualisation techniques utilised, and the inferences that may be drawn from the atlas estimates by researchers, managers, and policy makers, it is our aim here to outline the rationale and development of the statistical models used for the Australian Cancer Atlas, and the method of visualising the estimates from those models. In doing so, it is hoped that these statistical and visualisation methods may be used more widely, thus creating opportunities for more direct comparisons between the geographical patterns of cancer burden and possible causes or risk factors, both within Australia and across other countries.

## Methods

### Geographical areas

The geographical areas that compose the Atlas are Statistical Areas level 2 (SA2s), defined by the Australian Statistical Geography Standard (ASGS) July 2011 edition [13]. SA2s broadly represent communities that interact together socially and economically, and cover Australia without gaps or overlap [14]. Australian SA2s range in size from < 1 to 520,000 km^2^, and in 2014 had a median population of 8991 (range 0 to 54,773). There are two main reasons for using SA2s: they provide the best compromise between small population and small area to enable modelling at a “small area” scale; and they are the smallest geographical areas to which cancer registries routinely assign patient residence at diagnosis.

Of the 2214 SA2s covering Australia, those with no residential population (n = 28), no physical location (comprising ‘Migratory–Offshore–Shipping’ and ‘No usual address’ codes for each State and Territory) (n = 18), fewer than five residents on average per year during 2010–2014 (n = 17), and those very remote islands > 500 km from the Australian mainland [Christmas Island, Cocos Island and Lord Howe Island (n = 3)] were excluded. This left 2148 SA2s for which modelled estimates are provided in the Atlas.

### Cancer types and outcome measures

The cancer types, classified using the 2016 version of the International Classification of Diseases (ICD-10) disease code (Table 1), [15] were selected based predominately on those with higher numbers of diagnoses. Due to discrepancies between how the state and territory cancer registries assigned invasive status during the time period of interest (2005–2014), bladder cancer (ICD-10 C67) was excluded from the analysis. Breast cancer was restricted to females only, due to an insufficient number of breast cancers diagnosed among males for analysis. Due to reporting estimates by males, females and all persons separately, 50 unique sex-specific (males, females, all persons) cancer types were included in the analysis.

**Table 1:** Time period and data sparsity by type of cancer

Cancer type	Total diagnoses	Number of SA2s with 0 counts			Number of SA2s with 0 people at risk 2006–2014		
		M	F	P	M	F	P
2010–2014							
All cancers C00–C97, D45, D46, D47.1, D47.3–D47.5	615,192	29	42	26	21	31	18
Bowel cancer (C18–C20)	74,223	71	88	61	60	74	48
Lung cancer (C33–C34)	54,792	90	115	74	71	91	64
Melanoma of the skin (C43)	61,310	95	110	73	74	88	42
Breast cancer^a^ (C50)	77,992	–	62	–	–	46	–
Prostate cancer^b^ (C61)	99,681	57	–	–	41	–	–
2005–2014							
Head and neck cancer (C00–C14, C30–C32)	40,441	72	211	67	73	223	67
Oesophageal cancer (C15)	13,362	252	700	170	264	751	177
Stomach cancer (C16)	20,084	192	433	139	201	476	146
Liver cancer (C22)	14,985	244	660	172	264	728	186
Pancreatic cancer (C25)	26,939	162	223	103	176	253	106
Cervical cancer^a^ (C53)	7930	–	302	–	–	308	–
Uterine cancer^a^ (C54–C55)	22,020	–	101	–	–	105	–
Ovarian cancer^a^ (C56)	13,267	–	199	–	–	211	–
Kidney cancer (C64)	28,212	114	248	91	116	252	92
Brain cancer (C71)	14,933	255	392	156	269	418	165
Thyroid cancer (C73)	21,895	443	189	123	452	192	125
Non-Hodgkin lymphoma (C82–C86)	45,169	96	146	71	96	152	71
Myeloma (C90)	15,264	279	431	172	294	456	182
Leukaemia (C91–C95)	30,737	124	200	88	134	217	91

*M* males, *F* females, *P* persons

^a^ Females only

^b^ Males only note that only the 2148 SA2s considered to have a residential population are included

#### Incidence

Cancer incidence refers to the number of new cancer cases diagnosed within a given time period. In order to make comparisons of cancer incidence between small areas with differing population size and age structure, incidence rates were compared rather than the number of cancers diagnosed. Indirect standardisation through the standardised incidence ratio (SIR) [16] is the preferred method of standardisation when there are very small numbers in some age groups, because it removes the substantial sampling variation that would be present in this situation with direct standardisation [17]. The SIR reflects the area-specific incidence rate relative to the Australian average. It is the ratio of the observed cancer cases to the expected number of cases, the latter adjusting for differences in population between SA2s and differences in age structure of the population within an SA2.

#### Survival

Survival is a key measure of cancer patient care [18]. Consistent with standard reporting from population-based cancer registries [18], the relative survival framework was used in this Atlas. Relative survival is an estimate of net survival, which aims to measure the deaths that are specifically associated with a cancer diagnosis. For the Atlas, relative survival estimates up to 5 years after diagnosis were generated using the period method [19]. The relative survival models estimate the excess hazard ratio (EHR), which, for a given geographical area, is interpreted as the risk of dying from a given cancer within 5 years after diagnosis relative to the Australian average risk [18].

#### Time period

To maximise the robustness of the spatial estimates due to the sparse data (Table 1), the cancer cases were aggregated over multiple years. For cancer incidence, the five most commonly diagnosed cancers and the category “all cancers” used data aggregated over the latest 5-year period (2010–2014), while all other cancer types combined cases over the latest 10-year period (2005–2014). For the cancer survival estimates, data were aggregated across the entire ‘at-risk’ time period for which population mortality data were available, namely 2006–2014. Given that the period method was being used to calculate relative survival, cases included in the survival analyses could have been diagnosed from 2001 onwards.

### Data sources

De-identified individual level data for each case of cancer diagnosed in Australian residents during the specified period were obtained from the Australian Cancer Database (ACD), which is maintained by the Australian Institute of Health and Welfare (AIHW) [20]. The ACD contains records on all primary invasive cancer cases (excluding basal and squamous cell carcinoma of the skin) diagnosed in Australian residents and notified to one of Australia’s eight state and territory population cancer registries. Australia has mandatory cancer notification, ensuring virtually complete population incidence data. At the time of analysis, the ACD contained records of cases diagnosed from 1982 to 2014 (1982–2013 in New South Wales), although geographical information for cancer cases at the SA2 level was only available from 1996 onward. Cases with missing SA2 details (0.8% of all cancers) were excluded from the analyses.

Estimated residential population data grouped for Australia by SA2, year, sex, and 5-year age group (to 85+ years) were obtained from the Australian Bureau of Statistics (ABS) [21]. These population data were only available from 2001 onward.

Unit record population mortality data for all causes of death combined were only available from the Registry of Births, Deaths and Marriages [22] for the period during 2006 to 2014. The unit record data included details about sex, year of death, age, and SA2 at death.

### Statistical models

#### Spatial smoothing

Due to the inherent variation that is associated with low numbers in small geographical areas, spatial smoothing was used to increase the stability of the resulting estimates and protect confidentiality. In effect, spatial smoothing borrows information from neighbouring areas, seeking to estimate the underlying rate, rather than simply reporting the more pronounced random variation arising from very low numbers of observed cases when there are few residents. Spatial smoothing also alleviates the effect of the somewhat arbitrary nature of boundaries defining the geographical areas.

There are several ways to carry out statistical smoothing. For the Atlas we used Bayesian spatial models, in particular, conditional autoregressive models. Two main advantages of this approach were that Bayesian models readily incorporate the spatial correlation between areas in a natural manner as part of the prior information, recognising that adjoining geographical areas are likely to have some similar characteristics, and also that the probabilistic description of each of the unknown quantities provides a unique capacity to better quantify the extent of uncertainty around the spatial estimates.

The use of prior distributions to specify the unknown parameters is especially helpful for spatial parameters as it imposes a structure on the underlying stochastic process that is consistent with Tobler’s first law of geography, namely that “near things are more related than distant things” [23]. The resulting spatial smoothing, or shrinkage, pulls posterior estimates towards a local or global mean, improving the stability of the estimates, especially areas with small populations, thus providing more robust estimates [24, 25]. The extent of the smoothing depends on both the data and the specific prior distribution used.

Parametric Bayesian models provide a posterior distribution for the unknown parameters, not just a point estimate. This is helpful in understanding the uncertainty in the estimates [26, 27]. Estimates obtained from Markov Chain Monte Carlo (MCMC) sampling are particularly useful as several statistics such as credible intervals around the estimate and probability that the estimate reflects a real difference to the Australian average can be derived which quantify uncertainty from several different perspectives.

#### Spatial weights

A feature shared by all spatial prior distributions is the specification of spatial proximity between the random effects for each pair of areas. This usually takes the form of a spatial weights matrix, ${\mathbf{W}}$ [26, 28–30]. There are many ways to define spatial proximity, which can be either continuous, e.g. distance between areas, or discrete, e.g. belonging to the “neighbourhood” of adjacent areas. The most common definition is the binary, first-order, adjacency weights matrix which has elements

1 $$w_{ij} = \left\{ {\begin{array}{*{20}l} 1 \hfill & {{\text{if areas}} \;i\;{\text{and}}\;j\;{\text{are adjacent}}} \hfill \\ 0 \hfill & {\text{otherwise}} .\hfill \\ \end{array} } \right.$$

This is the specification used in the Atlas, where SA2s are considered adjacent if they share a common land boundary of any length (so a SA2 wholly enclosed within another SA2 is considered adjacent to that SA2 and thus only has one neighbour). Under this definition, there were 12 island SA2s without neighbours. To enable spatial smoothing for these areas, at least one non-adjacent area was assigned as a neighbour. This was usually the mainland area connected by a bridge or ferry service, or failing that, the closest mainland point.

Although different specifications of the weights matrix can impact smoothing and lead to different inference on the spatial patterns, weights based on a distance decay function are usually deemed more suitable when the areas vary greatly in shape and size [31, 32], or the population varies in size or age structure [33]. We chose binary adjacency weights for several reasons. First, alternative weights such as those based on distances between area centroids do not automatically induce the Markov property [34, 35], whereas a sparse spatial weights matrix is computationally advantageous. Second, distance-based weights with a high rate of decay as is commonly seen in disease atlases tend to become similar to row-standardised, binary, adjacency weights [36], which seemed counterproductive. Conversely, a low rate of decay increases the region of influence implied by the spatial weights matrix, which may imply unrealistic spatial dependencies between the areas [36]. Fewer neighbours were not only computationally advantageous, but also tended to perform at least as well as models with more neighbours [29, 33, 36]. Given the vast differences in the size of SA2s in Australia, we also found that distance-based weights, with a suitable cut-off to retain the Markov property, resulted in excessively large or small regions of influence. Third, adjacency-based weights complement the discrete nature of the observed data [30], and grant the straightforward interpretation of including the average response values of the neighbouring areas as an extra predictor [28]. Fourth, adjacency-based weights are easy to implement and alternative definitions do not necessarily improve results [29, 33]. Moreover, an investigation of different spatial models (see Cramb et al. [37]) and sensitivity analyses conducted as part of the development of the Atlas indicate that the type of model and the hyperprior controlling the variance of each spatial random effect has a greater influence on smoothing than the spatial weights.

#### Bayesian hierarchical models

The models chosen for cancer incidence, and to some extent, relative survival, were based on the recommendations from Cramb et al. [37]. In addition to the choice of neighbourhoods via the spatial weights, described above, a range of smoothing approaches across the nominated neighbourhoods are possible. Models may employ the same smoothing parameter consistently over the entire region (‘global smoothing’) or allow it to vary geographically based on specific characteristics such as geographical, social and administrative measures (‘local smoothing’). Furthermore, there are choices to be made about the underlying model, the sampling distribution to use for the data, the inclusion of fixed and/or random effects, and the specification of the prior distributions for the unknown parameters. A range of smoothing approaches and also model forms were considered for the Atlas, including parametric and semi-parametric models. The final choice was made by comparing the performance of each model and spatial prior configuration across a range of criteria, including goodness-of-fit, computation time, tendency to under- or over-smooth, and plausibility of estimates.

The model chosen for both cancer incidence and relative survival was that proposed by Leroux et al. [38]. The Leroux model was favoured over other spatial models including the popular BYM model [39] because it provided the best compromise between the aforementioned criteria [37]. This model is a specific case of the generic three-stage hierarchical model proposed by Best et al. [40] for the purpose of disease mapping.

##### Incidence model

The cancer incidence model is given by,

$$\begin{aligned} & y_{i} \sim {\text{Poisson}}\left( {E_{i} e^{{\theta_{i} }} } \right)\quad {\text{for}} \;i = 1, \ldots ,2148 {\text{SA}}2{\text{s}} \hfill \\ & \quad \theta_{i} \sim \beta_{0} + S_{i} \hfill \\ \end{aligned}$$

where $\varvec{y}_{\varvec{i}}$ is the observed number of cases for the $i{\text{th}}$ area, $E_{i}$ is the expected number of cases, $\theta_{i}$ is the log-SIR, $\beta_{0}$ is the overall level of log-SIR (a fixed effect), and $S_{i}$ is the spatial random effect modelled by the Leroux prior

2 $$S_{i} |\varvec{S}_{\backslash i} \sim {\mathcal{N}}\left( {\frac{{\rho \mathop \sum \nolimits_{j} w_{ij} S_{j} }}{{\rho \mathop \sum \nolimits_{j} w_{ij} + 1 - \rho }}, \frac{{\sigma_{S}^{2} }}{{\rho \mathop \sum \nolimits_{j} w_{ij} + 1 - \rho }}} \right)$$

Here $w_{ij}$ are the elements of the spatial neighbourhood matrix defined in Eq. (1), and $\rho$ determines the spatial autocorrelation between the areas. The modelled estimates are effectively age-adjusted since the expected counts take into account the population size and age-structure. The prior distributions for the remaining parameters were weakly informative, specifically,

$$\begin{array}{*{20}c} {\beta_{0} \text{ }\sim { \mathcal{N}}\left( {0, 10^{5} } \right)} \\ {\sigma_{S}^{2} \text{ }\sim { \mathcal{I}\mathcal{G}}\left( {1, 0.01} \right)} \\ {\rho \sim {\text{Uniform}}\left( {0, 1} \right).} \\ \end{array}$$

where the inverse Gamma distribution ${\mathcal{I}\mathcal{G}}\left( { \cdot ,\text{ } \cdot } \right)$ is parameterised in terms of shape and scale. See the section “Sensitivity analyses” for further discussion of these priors. The key output from this model and reported in the Atlas is the SIR, given by ${ \exp }\left( {\theta_{i} } \right)$.

##### Relative survival model

The relative survival model is closely based on that proposed by Fairley et al. [41] except that the spatial random effect was modelled by the Leroux prior rather than the BYM prior [39]. The likelihood for the number of deaths observed for the $i{\text{th}}$ area in the $k{\text{th}}$ strata and $t{\text{th}}$ follow-up interval is Poisson,

$$d_{itk} \sim {\text{Poisson}}\left( {\mu_{itk} } \right)$$

for $i = 1, \ldots ,2148$ areas, $k = 1, \ldots ,K$ age-sex-site strata, and $t = 1, \ldots ,5$ follow-up years. The value of *K* depends on the cancer being modelled, but in general, it accounts for broad age groups (15–54, 55–64, 65–74 and 74–89 years), and for all persons only, sex (males, females). These broad age groups were chosen based on the frequency distribution of the observed cancers diagnosed, with the intention of relatively equal numbers of cancers within each age group. The alternative option, equal-spaced age ranges, would have increased the sparsity of data within younger ages, so was not considered further. In addition, for the aggregated cancer groups of “all cancers combined” and “head and neck cancers”, *K* also included cancer site.

The expected number of deaths due to any cause, $\mu_{itk}$, is then modelled using the link function,

$$\log \left( {\mu_{itk} - d_{itk}^{*} } \right) = \log \left( {y_{itk} } \right) + \alpha_{t} + \beta_{1} x_{1,itk} + \cdots + \beta_{K} x_{K,itk} + S_{i}$$

where $d_{itk}^{*}$ is the expected number of deaths due to causes other than the cancer of interest, $y_{itk}$ is an offset parameter for person-time at risk, $\alpha_{t}$ is a year-specific intercept, $x_{1,itk} , \ldots ,x_{K,itK}$ are indicator variables relating to the age–sex-site strata. The spatial random effect, $S_{i}$, is modelled by the Leroux prior, Eq. (2), as it was for the incidence model, except the variance $\sigma_{S}^{2}$ was assigned a half-Normal prior

$$\sigma_{S}^{2} \sim {\mathcal{N}}\left( {0, 5} \right){\text{II}}_{{\left( {0,\infty } \right)}}$$

instead of an inverse Gamma distribution, where the indicator function II${\text{II}}_{{\left( {0,\infty } \right)}} = 1$ if $0 < \sigma_{S}^{2}$. The priors for the intercept, regression coefficients, and spatial smoothing parameter $\rho$ were assigned weakly informative priors, namely,

$$\begin{array}{*{20}c} {\alpha_{t} \text{ }\sim { \mathcal{N}}\left( {0, 1000} \right)} \\ {\beta_{k} \text{ }\sim { \mathcal{N}}\left( {0, 1000} \right) \quad {\text{for }}k = 1, \ldots ,K} \\ {\rho \sim {\text{Uniform}}\left( {0, 1} \right).} \\ \end{array}$$

The rationale for the choices of these priors is explained in further detail below (see “Sensitivity analyses”) The key output from this model and reported in the Atlas is the EHR, given by $\exp \left( {S_{i} } \right)$.

##### Uncertainty and posterior probability differences

The statistical models not only smooth the estimates spatially so as to provide more realistic estimates of the cancer burden in Australia, but they also reduce the uncertainty around these estimates. This is especially true when the observed cases are zero, effectively yielding an observed SIR, i.e. $y_{i} /E_{i}$, with unbounded uncertainty about the true value (keeping in mind that the residential population is at least 5, and that the SIR is modelled on the log-scale). This is true for the EHR too, at least conceptually. Presenting the uncertainty in the Atlas is important since similar point estimates with different levels of uncertainty can lead to different inferences.

In the Atlas, two different aspects of uncertainty are examined. The first aspect is the precision of the posterior point estimates of incidence and relative survival, which is quantified using credible intervals (CrIs), specifically 60% and 80% CrIs. Providing two credible interval widths gives greater information, and 80% is recognised as an appropriate choice for Bayesian models [42].

The second aspect relates to the probability that an estimate is different from the Australian average, taking into account its uncertainty. The posterior probability that the estimate in the $i{\text{th}}$ area is greater than 1 is given by

$$PP_{{i,{\text{high}}}} = \frac{1}{M}\mathop \sum \limits_{m = 1}^{M} {\text{II}}\left( {A_{i}^{\left( m \right)} > 1} \right)$$

where $A_{i}^{\left( m \right)}$ is the $m{\text{th}}$ MCMC estimate (SIR or EHR) for the $i{\text{th}}$ area. By symmetry, the probability of the $i{\text{th}}$ area’s estimate being less than 1 is $PP_{{i,{\text{low}}}} = 1 - PP_{{i,{\text{high}}}}$. Therefore, the confidence that the estimate is substantively different to 1 is given by $\left| {PP_{{i,{\text{high}}}} - PP_{{i,{\text{low}}}} } \right|$ which is equivalent to the scaled probability $2\left| {PP_{{i,{\text{high}}}} - 0.5} \right|$, referred to as the difference in posterior probabilities (DPP).

### Computation

An increasing range of algorithms is available for spatial models. Popular examples include MCMC simulation [43] and approximations such as integrated nested Laplace approximation (INLA) [44]. For the Atlas, we chose an MCMC approach to estimate the posterior distributions because it is easier to quantify the uncertainty of the posterior estimates compared to alternative approaches like INLA. This grants practical advantages in estimating comparative statistics and visualising the uncertainty as discussed below. The incidence models were implemented in R [45] using the CARBayes package v5.0 [46] while the relative survival models were implemented in WinBUGS [47] interfaced with R using the R2WinBUGS package [48].

**Fig. 1:**
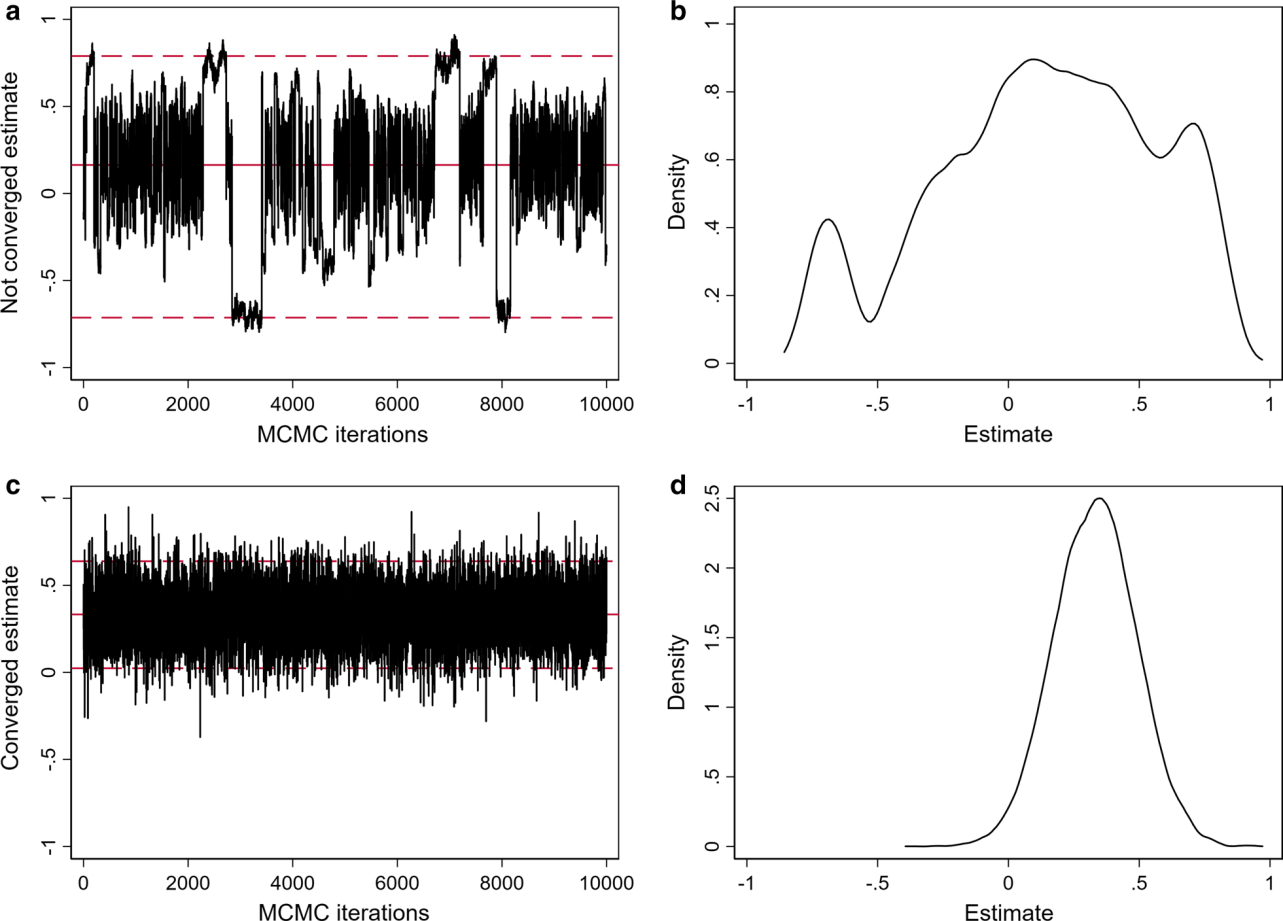
Example trace and density plots used to assess convergence of the MCMC chains for the log-SIR for two selected SA2s: **a** trace plot and **b** density plot for a model run on simulated data showing an example of lack of convergence; and **c** trace plot and **d** density plot for the Leroux incidence model for one selected cancer and SA2, showing convergence of the estimates used in the Atlas

For both incidence and relative survival models, the MCMC chains had a burn-in of 50,000 followed by an additional 100,000 iterations, keeping every 10th iteration to reduce dependence between samples, resulting in a posterior sample size of 10,000. Convergence of these chains was assessed by visual inspection of trace and density plots for selected parameters and areas (Fig. 1), and more formally using the Geweke diagnostic test [49]. Areas with a Geweke p-value < 0.01 were flagged to be examined visually for evidence of convergence. These tests indicated that the burn-in was sufficient.

### Sensitivity analyses

The priors for the incidence model correspond to the default priors for those parameters in CARBayes. The same priors were initially used for the regression coefficients and spatial smoothing parameter in the relative survival model, but subsequently made less vague due to convergence issues.

Moreover, a sensitivity analysis was conducted to determine how influential the priors were on the posterior estimates. Our analysis revealed that the estimates were insensitive to the prior choices, except for the prior on the variance of the spatial random effects, $\sigma_{S}^{2}$, for the relative survival model which tended to be more influential when the data were more sparse (i.e. for rarer cancers). For the incidence models, the sensitivity analyses for $\sigma_{S}^{2}$ revealed that the estimates were fairly robust to this prior choice.

Over 50 different prior specifications were considered for $\sigma_{S}^{2}$ in the relative survival model, including gamma, inverse gamma, and half-Normal distributions with different parameter values. Notable examples include the ${\mathcal{I}\mathcal{G}}\left( {1, 0.01} \right)$ prior for comparison to the incidence model, and half-Normal priors corresponding to Normal priors with variances of 3, 5, 7.5, and 10 each truncated at zero. The inverse gamma prior was too vague, leading to under-smoothing, as was the half-Normal prior with a variance of 10. The other three half-Normal priors gave reasonable results, showing slight variations in the degree of smoothing, so the ${\mathcal{N}}\left( {0, 5} \right){\text{II}}_{{\left( {0,\infty } \right)}}$ prior was chosen as a compromise between conservative estimates and estimates which will show genuine differences between areas.

### Level of evidence for spatial variation

This was assessed using Tango’s Maximised Excess Events Test (MEET) global clustering test [50], which has been shown to perform well across a variety of datasets [51]. The input data required for this test is the modelled counts (i.e. from the model results, the number of diagnoses or excess deaths per area) and the expected counts (as input into the Bayesian model). As it is expected to consider up to half the total area, our maximum distance of examination was 2000 km. The p-value from Tango’s MEET was divided into four categories, consistent with previous analyses [52], being strong (p-value < 0.01), moderate (p-value 0.01 to < 0.05), weak (p-value 0.05 to < 0.10) and none (p-value 0.10+). Tango’s MEET uses Monte Carlo methods, so to help ensure our categorisation was appropriate, we calculated Tango’s MEET three times, reporting only the most conservative category. Only one cancer and sex combination had different categories for diagnoses, and four for excess deaths. If there is one bar or less for the level of evidence, this indicates that overall there is no meaningful evidence that the estimate for this cancer type and sex varies across the country. There may still be some individual areas that differ from the national average, but given the lack of evidence for overall variation, these individual differences should be interpreted with greater caution.

### Visualisation

The Atlas consists of several visual components. The main component is the map of SA2s shaded according to point estimates of the SIR/EHR. Also included are visualisations which clarify the uncertainty around those estimates and whether they represent real differences to the Australian average, and visualisations to better illustrate spatial patterns from a national perspective. At the start of the Atlas development, we initiated an independent scoping process to obtain, among other things, a consensus about the key users for the Atlas, and then combined these into four target groups—the general public, policy makers, scientific researchers and health practitioners. When designing the presentation of the results from the statistical analysis in the Atlas, consideration was given to why each group would use the atlas and their skill levels. Feedback from people within these user groups was obtained during the development of the Atlas through focus groups and stakeholder workshops.

#### Maps

For each SA2, the models generate a posterior distribution each of the unknown parameters, including the two main quantities of interest, the $SIR_{i} = \exp \left( {\theta_{i} } \right)$ and $EHR_{i} = \exp \left( {S_{i} } \right)$. To avoid any undue influence from outliers, the median of the 10,000 monitored MCMC iterations is used in the maps.

##### Colour schemes

The estimates $SIR_{i}$ and $EHR_{i}$ for a given area were intended to be interpreted in relation to the Australian average, which by construction, has an SIR and EHR of 1. To facilitate this interpretation at a national level, so that the spatial patterns of cancer, if any exist, are easily observable on the Atlas maps, the estimates were mapped according to a diverging colour gradient where yellow represents the Australian average, darker shades of orange/red indicate SA2s with an estimate above the Australian average (higher than average risk), and shades of blue indicate SA2s with an estimate below the Australian average (lower than average risk). Both SIRs and EHRs were mapped to the same spectrum of colours to facilitate comparison of spatial patterns between incidence and survival estimates. The colour gradients were linear on the log scale, with the darkest red reflecting an SIR of 1.5 (a 50% higher risk of diagnosis or death within 5 years than the average) or greater, and dark blue beginning at the inverse point (≈ 0.67). In making the colour selection, we considered the needs of people with various forms of colour blindness. A Colour Blindness Simulator (https://www.color-blindness.com/coblis-color-blindness-simulator/) provided substantial evidence that the chosen colour scheme was still interpretable by most forms of colour blindness, apart from monochromatism.

##### Transparency layer

**Fig. 2:**
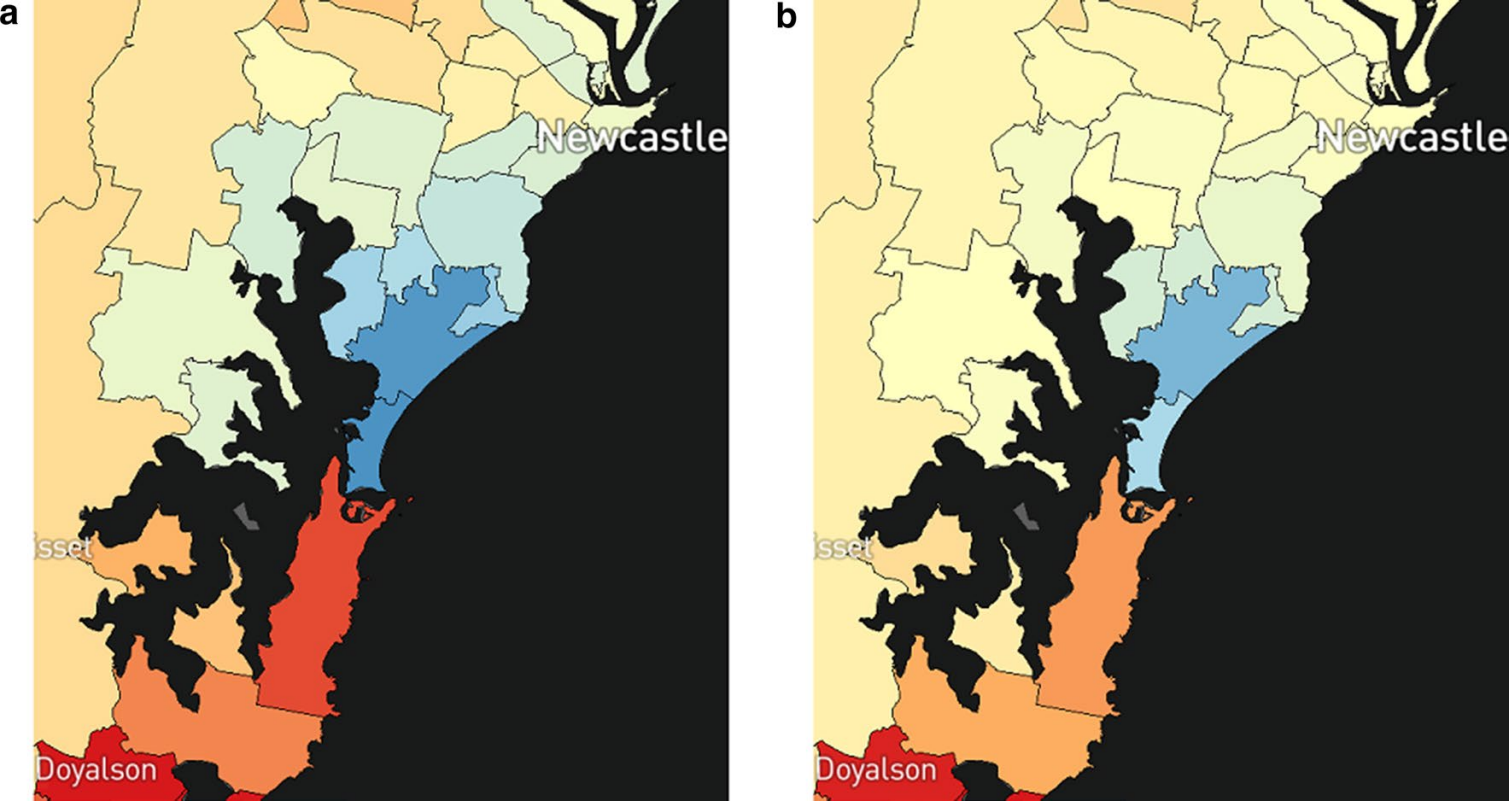
Partial map of liver cancer incidence for females with **a** transparency overlay disabled, and **b** transparency overlay enabled (default option)

So that imprecise estimates do not give the impression of important differences, a second layer with varying degrees of opacity was also developed and displayed (Fig. 2). This has the pale-yellow colour of the Australian average in increasing opacity (0% if an estimate has a DPP of 1, 100% if an estimate has a DPP of 0), so that estimates with very high uncertainty were less visually distinguishable from the Australian average, regardless of their median values.

#### Graphs

##### Summary plots for large regions

**Fig. 3:**
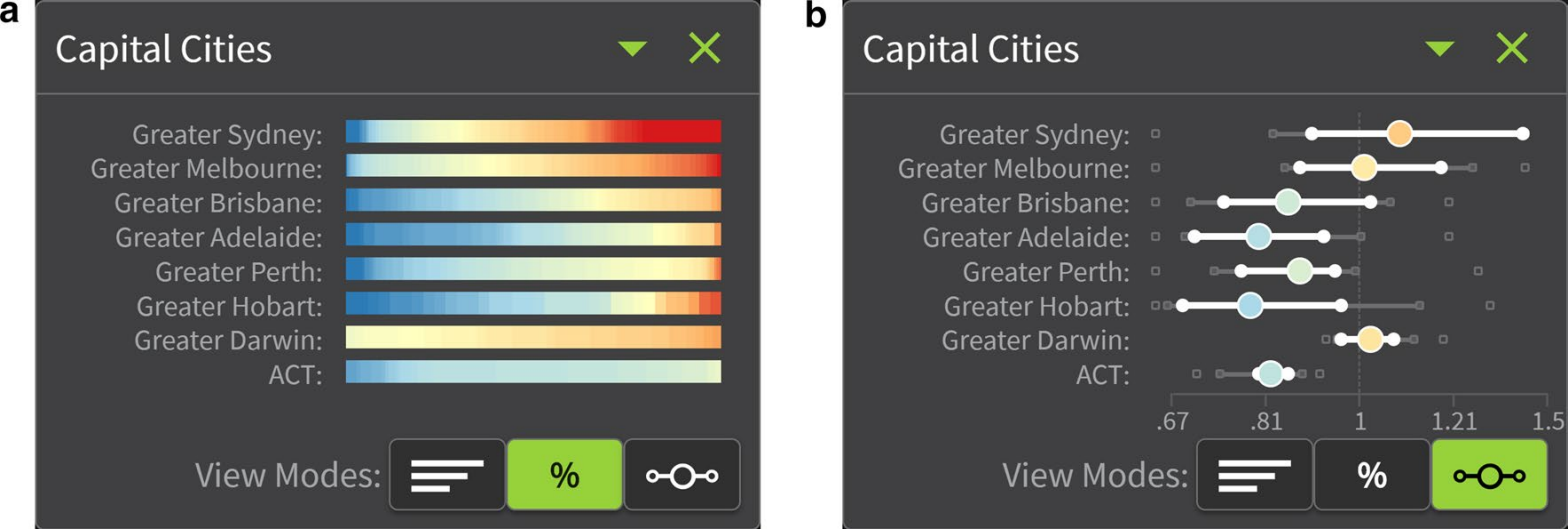
Example of the summary plots for large regions, showing liver cancer incidence for females by SA2 as **a** a percentage of SA2-specific estimates, and **b** a distribution (boxplot) of SA2-specific estimates

The nationwide patterns can be difficult to visualise geographically due to small SA2s, especially in urban areas, which are not visible without zooming in. To overcome this, summary plots showing the distribution of SA2-specific estimates were developed so that all of Australia could be represented, either in its entirety (quintiles of area-based socioeconomic groups using the Index of Relative Socio-economic Advantage and Disadvantage [53], remoteness areas (categories of the physical distance of a location from the nearest areas of concentrated urban development with populations of 200 people or more) [54], or state and territory boundaries), or focused on regions which were difficult to see on the map (greater capital city areas [55]). An example is shown in Fig. 3.

##### V-plots

**Fig. 4:**
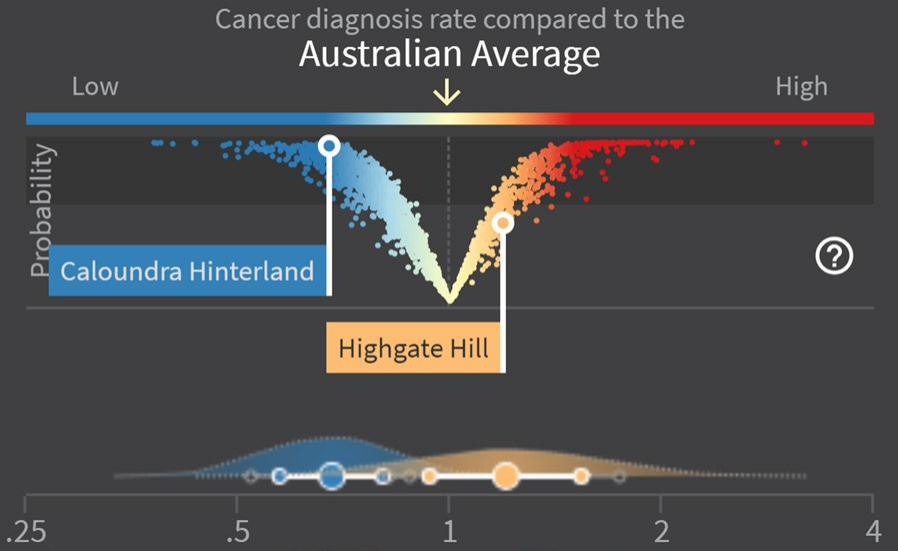
Example of the V-plot and wave plot showing liver cancer incidence for males for two selected SA2s

A V-plot (Fig. 4) was developed as a new method for combing two pieces of information, namely the area-specific SIR/EHR estimates and the probability that these estimates are different from the Australian average. The x-axis compares the posterior median SIR/EHR of each area to the Australian average, while the y-axis is the posterior probability that the true incidence/relative survival for an area is different from the Australian average; this is given by the DPP. Estimates near the top of the V-plot are likely to reflect a real difference from the Australian average, while estimates near the bottom of the V-plot are unlikely to be a real difference.

##### Wave plots

The precision around a posterior estimate of the SIR (reflected by the 60% and 80% CrIs), was summarised visually by a plot of the empirical density for the logarithm of the SIR, where the labels were adjusted to reflect the scale of the SIR (Fig. 4). Similarly for the EHR. The representation on the log-scale addresses the problems associated with densities for ratio scale parameters, most notably the apparent differences in the area under the curve resulting from the non-linear intervals. Since these densities are not true densities, but are analogous in their interpretation, we refer to them as “wave plots”.

**Fig. 5:**
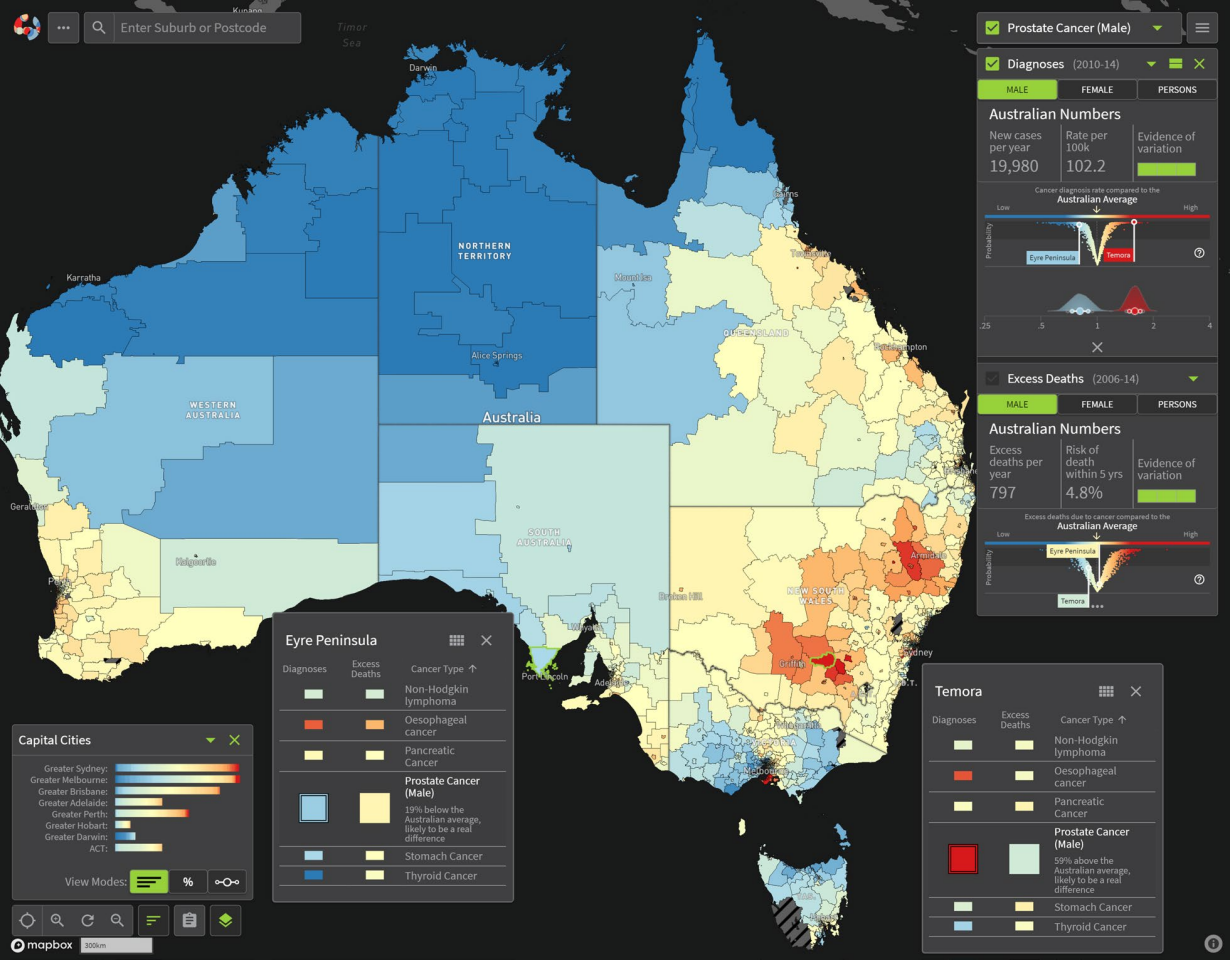
Example view of the Australian Cancer Atlas showing the geographic variation of prostate cancer incidence

## Results

The results of the statistical model and visualisation development were published through the release of the Australian Cancer Atlas (https://atlas.cancer.org.au) in September, 2018. The Australian Cancer Atlas provides the first freely available, digital, interactive picture of cancer incidence and survival at the small geographical level across Australia with a focus on incorporating uncertainty, while also providing the tools necessary for accurate estimation and appropriate interpretation and decision making. The main user interface of the Atlas is illustrated in Fig. 5.

## Discussion

The Australian Cancer Atlas is the first comprehensive, interactive digital cancer atlas based on small areas and utilising spatial smoothing to describe geographical patterns of cancer incidence and survival across Australia. One of the key objectives of the Atlas was to motivate new research to gain a better understanding of why geographical variation exists. Its success will be measured in part by how widely the Australian Cancer Atlas is used by key stakeholders, including members of the community, researchers, clinicians, and government, both as a source of information and to guide research and inform decision making.

Towards this aim, we plan an ongoing program of development and research. Future versions of the Atlas are planned to incorporate information about how geographical patterns have changed over time, new indicators such as cancer screening, diagnostic tests, and cancer treatment, and alternative visualisations such as cartograms which alleviate overemphasis of spatial patterns exhibited by large but lesser populated areas.

It is also hoped that the Atlas will provide motivation and opportunity for a better understanding of why geographical variation exists for each type of cancer. These investigations will need to consider in more detail the characteristics of people living within areas, the stage and other clinical characteristics of the cancer, treatment patterns, and the distribution of known cancer risk factors and cancer screening behaviour. They will also incorporate ecological modelling to identify associations between key area-level factors and geographical patterns such as remoteness, area disadvantage and access to services. A multidisciplinary approach will be crucial to meet these goals, and it is hoped that the Atlas provides motivation and opportunity for different research groups to collaborate and combine existing data at the small area level.

## Conclusions

Since its release in September 2018, the Australian Cancer Atlas has brought new insights about geographical cancer patterns across Australia. It has already motivated new collaborations within Australia and internationally to better quantify and understand the geographical patterns of cancer indicators, and will continue to form the foundation of an ongoing research program incorporating innovative statistical models and visualisations to better quantify the extent and characteristics of the geographical variation, and the reasons why it exists.

## Data Availability

The datasets generated during the current study are available for download via the Australian Cancer Atlas (https://atlas.cancer.org.au). The original data that support the findings of this study are available from the Australian Cancer Database, Australian Institute of Health and Welfare, but restrictions apply to the availability of these data, which were used under license for the current study, and so are not publicly available. With appropriate ethics and data custodian approvals, these data can be obtained from the Australian Institute of Health and Welfare.

## Abbreviations

ACD: Australian Cancer Database
ASGS: Australian Statistical Geography Standard
CAR: conditional autoregressive
CrI: credible interval
DPP: difference in posterior probabilities
EHR: excess hazard ratio
ICD: International Classification of Diseases
MCMC: Markov Chain Monte Carlo
MEET: Maximised Excess Events Test
SA2: Statistical Area 2
SIR: standardised incidence ratio
